# “*We're going on a virtual trip!*”: a switching-replications experiment of 360-degree videos as a physical field trip alternative in primary education

**DOI:** 10.1186/s40723-023-00110-x

**Published:** 2023-01-25

**Authors:** Manuel B. Garcia, Louis S. Nadelson, Andy Yeh

**Affiliations:** 1 Educational Innovation and Technology Hub, FEU Institute of Technology, Manila, Philippines; 2 College of Education, University of Central Arkansas, Conway, AR USA; 3 Faculty of Creative Industries, Education and Social Justice, Queensland University of Technology, Brisbane, QLD Australia; 4 College of Education, University of the Philippines Diliman, Quezon City, Philippines

**Keywords:** Virtual field trip, 360-degree videos, Immersive videos, Primary education, Experimental design

## Abstract

Field trips are steadily declining due to limited funding, time constraints, safety concerns, and other logistical issues. Many schools are resorting to a virtual field trip (VFT), especially when education is disrupted due to public health concerns, natural disasters, or other unforeseen significant events. Virtual reality as a common form of VFT is likely not an option for many schools due to cost and other barriers. The purpose of our study was to explore the potential of going in a VFT using 360-degree (360°) videos as an alternative to a physical field trip in primary education. We recruited third-grade pupils (aged 8–9) from two private elementary schools to experience VFTs using 360° videos (360V) and regular videos (REGV). Using a switching-replications experimental design, we compared their content recall (assessment tests) and VFT experience (attitude, perceived usefulness, involvement, inquiry, video engagement, and virtual guide) across four-time points. Our results show that the increase in content recall scores of 360V groups after VFTs was consistently higher compared to REGV groups at all time points, although it was only significant in one quarter. We also found pupils’ video engagement, involvement, and attitude as significant factors in their VFT experience. These results call attention to a possible implementation of VFTs and continue the long-standing tradition that has been acknowledged as a student-centered, interactive instructional method.

## Introduction

A field trip is a school excursion that extends student learning beyond the classroom by exploring new environments. It is a long-standing tradition that has been acknowledged as an integral student-centered, interactive instructional method (Behrendt & Franklin, 2014). Under the auspices of the school, teachers and students embark on a journey outside the school building to develop an experiential connection between the concepts and ideas presented in various subject matters. Such on-site field experience offers students an authentic encounter with a natural environment, thereby becoming aware of the real world and its association with their classroom learning. This practical activity helps them in decoding the complex knowledge they are being taught in school (Bowker & Tearle, 2007; Kola‐Olusanya, 2005; Lebak, 2007; Skop, 2009). Moreover, field trips allow students to develop a greater vocabulary, an increased perception of learning, and a heightened interest in the outdoors (Hoisington et al., 2010). Researchers such as Knapp and Barrie (2001), Hudak (2003), Kisiel (2006), and Nadelson and Jordan (2012) have investigated knowledge acquisition and the permanent change in attitudes and behaviors of students that occurred during field trips. Their studies emphasized that well-organized field trips aligned with the school curriculum and designed to meet specific educational objectives resulted in successful cognitive, affective, and psychomotor learning outcomes.

Despite its many potentials, field trips are steadily declining and many educational institutions do not organize such experiential activities as much as before anymore. The common reasons for the disappearance of field trips include limited funding, time constraints, safety concerns, logistical issues, and difficulty controlling student behavior (Behrendt & Franklin, 2014; DeWitt & Storksdieck, 2008; Higginsa et al., 2012). As a cost-free alternative, Behrendt and Franklin (2014) recommended campus field trips to maintain the benefits of a physical field trip (PFT). Although this type of field trip may be a viable alternative, it may be curtailed due to situations like COVID-19 in which schools are shuttered as a safety measure (Garcia, 2022; Pokhrel & Chhetri, 2021). To continue the tradition and ensure students acquire experiences that cannot be duplicated in the classroom, many schools are resorting to its virtual version (e.g., Evelpidou et al., 2021; Han, 2020; Seifan et al., 2020; Springer et al., 2020). Also titled virtual field guide or virtual excursion, a virtual field trip (VFT) is the exploration of digital worlds with the same educational intent as the PFT. It can take place in a range of digital platforms, such as single site exploration, curated collections (e.g., museum websites), or general exploration of the internet, which may be less structured and more self-determined by the student. In addition, the technology used may also vary, including the use of augmented reality (AR) and virtual reality (VR). However, the potential benefit of immersing students in augmented or virtual worlds may be limited due to issues of access, cost, and other barriers. In the absence of a VR headset, Rupp et al. (2019) reported that it is better to use a non-VR device (e.g., smartphones) than a low-fidelity consumer VR (e.g., Google Cardboard). The latter increases simulator sickness, particularly on the disorientation and oculomotor subscales, making the user experience uncomfortable and thereby limiting the consideration of low-cost options.

Fortunately, the recent advancements in video technologies offer exciting new modalities to implement a VFT. An example of new technology is 360-degree (360°) video. The educational research on 360° videos is still in its infancy, as shown by a systematic literature review (Ranieri et al., 2020). Thus, the cognitive impact and student experience with a VFT need to be documented. In addition, most research on 360° videos focused on higher education, retaining a dearth of research on 360° videos used by students in primary education. Our research addresses the gap in the literature by exploring the possibility of using 360° videos as an alternative to PFTs in primary education. To measure the applicability of the platform, we adopted a switching-replications experimental design to compare 360° videos (360V) with regular videos (REGV) as a mode of VFT across four-time points. We also examined the students’ content recall (assessment tests) and their perceptions of their VFT experience (attitude, perceived usefulness, involvement, inquiry, video engagement, and virtual guide). Our design addressed another literature gap by assessing the longer-term impact of school field trips (DeWitt & Storksdieck, 2008). Further, most of the VFT research we reviewed combined 360° videos with VR technology. Thus, it is unclear whether existing findings translate to using 360° videos alone, which are less immersive than 360° videos powered by VR platforms (Rupp et al., 2019).

## Background of the study

### VFT: a replacement for physical field trips?

In the early experimentation with student engagement in a VFT, Spicer and Stratford (2001) found students considered the virtual option as an enjoyable way to learn but did not think the virtual experience could be a substitute for a real field experience. Rather, they regarded VFTs as an enhancement to their fieldwork that offers a valuable indirect field experience and a way to empower physically or financially disadvantaged students (Stainfield et al., 2000). Prior VFT research involved using computers and digital visuality, such as hypertexts, videos, sound clips, and photographs, whereby students were passively browsing, watching, listening, and observing. An example is a VFT to Cumberland Island National Seashore (Hosticka et al., 2002) in which students passively observed someone else’s actual field experience. Technology advances have led to increased interest in VFT usage in education, especially with the new opportunity for students to actively interact with the virtual world (Springer et al., 2020). In addition, situations like the COVID-19 pandemic have prompted the reimagination of traditional field trips, making the VFT experience a more viable and acceptable alternative (Thönnessen & Budke, 2021).

Recent studies demonstrate that more technological advancements may be needed for VFTs to be widely accepted as equivalent to PFTs (Evelpidou et al., 2021; Seifan et al., 2020). Instead of serving as a replacement, VFTs are currently most commonly considered a supplement to PFTs that provide necessary pre-information before students visit an actual location (Seifan et al., 2019) or after an in-person field trip for better recall of experience (Harron et al., 2019). Çaliskan (2011) also asserted that VFTs are useful when certain locations cannot be visited due to time, safety, weather, or other constraints. For instance, Evelpidou et al. (2021) designed a VFT to Corinthian Gulf with five stops (i.e., Cenchreae, Lechaeum, Lake Vouliagmeni, Diolkos, and Heraeon), a trip that would be prohibitive for most students. The authors also used the VFT to disseminate geoarchaeological and geomorphological information. A similar VFT was conducted by Jitmahantakul and Chenrai (2019) where 360° VR environments were created to showcase landscapes and geological features in three dimensions. The current restrictions on public mass gatherings due to public health concerns (e.g., the spread of COVID-19), VFTs are positioned as viable replacements rather than its previous supplementary role to PFTs. Until such time that there are no more threats for the education sector to pursue returning to normal operations, VFT will serve as the primary means of conducting field trips across educational levels.

### The prevalence of 360° videos in education

Over the years, the use of video materials for teaching and learning has gained acceptance by teachers and students (Noetel et al., 2021). Many facets of education delivery (i.e., traditional, online, and hybrid) often include video integration for instruction. The wide array of instructional uses of videos include live streaming of lessons (Huang & Hong, 2017), playing video clips as part of the lecture (Kosterelioglu, 2016), and uploading recorded video lectures as a methodology for asynchronous education (Garcia, 2022). With the continued advancements in video technologies, the extent of its pedagogical implementation is steadily increasing—with 360° video as one of its latest innovative forms. Also referred to as immersive videos, 360° video is a type of video content recorded in an omnidirectional form allowing viewers to control viewing direction while watching. Video recordings are captured using special camera equipment (e.g., *Samsung Gear 360*, *Nikon KeyMission 360*, and *Kodak PIXPRO SP360*) and edited (or stitched together) in post-production using standard video-editing software. The videos are still viewable using regular video players, which also have additional drag and drop functionality for panning the viewing perspective. Since viewers of 360° videos can use a mouse or keyboard to roll, pitch, and yaw to explore different parts of the scene, these videos are more immersive than traditional 2D videos albeit less immersive than 360° videos in a VR platform (Rupp et al., 2019).

Quite reasonably, there has been an increasing prevalence of 360° videos in education (most often with a combination of VR technology). Some pedagogical applications of 360° videos are cultural heritage virtual tours (Argyriou et al., 2020), supplemental materials in laboratory experiments (Ardisara & Fung, 2018), viewing modality of medical procedures (Arents et al., 2021), and safety skills teaching tool (Araiza-Alba et al., 2021). Most of the research on using 360° videos for instruction report no significant difference in student learning outcomes compared to traditional instructional approaches. For instance, comparing students’ knowledge recall between a conventional education group and the 360° VR video group in an Obstetrics and Gynecology internship curriculum revealed no significant difference (Arents et al., 2021). Similarly, teaching water-safety skills to children did not significantly differ whether using either traditional teaching mediums or 360° (Araiza-Alba et al., 2021). Both studies suggest that 360° videos are more effective in the affective rather than cognitive domain. Meanwhile, the 360° immersive video virtual tour developed by Argyriou et al. (2020) for the historical city of Rethymno, Greece elicited a high level of user engagement and a satisfying immersion experience. This result can be attributed to the design of the virtual tour, which was a strategic combination of experience (e.g., the flow of the story) and interaction (e.g., navigation in the virtual world). The results reinforce the importance of being mindful of the different facets of 360° videos that can influence learner engagement and knowledge acquisition.

## Methodology

### Research design

We structured our research using a switching-replications experimental design in which an experimental (360V) and a control group (REGV) switched roles throughout the four rounds of experimentation. Customarily, this research design features only two rounds of the experiment (Trochim & Donnelly, 2008). We adjusted our experiment setup into four rounds to match the number of quarters in an academic year. In each quarter, we designated themes to follow the concept of a traditional field trip where multiple locations are visited. We consulted with the schools and they approved the selected themes before we carried out the interventions. The variation of themes and locations was part of our strategy to ensure that each group not only has more than one but also a unique VFT experience per intervention. Following this format, we performed an experiment before the end of each quarter of the academic year 2020–2021. See Table 1 for the sequence of the experiment themes and interventions.

**Table 1:** Sequence and experiment themes

Quarter	VFT themes/locations	Group 1 (G1)	Group 2 (G2)
First (Q1)	Farms and factories	360V	REGV
Second (Q2)	Museums and galleries	REGV	360V
Third (Q3)	Zoos and wildlife parks	360V	REGV
Fourth (Q4)	Historical sites and landmarks	REGV	360V

Our design is not directly aligned with the structure of traditional field trips, which customarily happen once a year. We deviated from the traditional structure to strengthen the statistical power and control for threats to internal validity (Edmonds & Kennedy, 2017). This design modification allowed us to address the loss of control over essential resources (e.g., physical access to experimental settings) caused by the COVID-19 pandemic, which posed risks to internal validity and generalizability (Alsiri et al., 2021). Furthermore, we adopted this research to eliminate the necessity of denying participants (control group) a possibly beneficial treatment caused by random assignment. During these trying times when schoolchildren are confined within their homes, we believe that everyone deserves access to any intervention strategy. Moreover, while the random assignment is considered to be the most robust method for determining the impact of a particular treatment (Alferes, 2012; Shadish & Ragsdale, 1996), it is rarely possible in educational research (Davies et al., 2008). Fortunately, Edmonds and Kennedy (2017) asserted that a switching-replications experimental design works well for researching educational interventions where learning events are repeated at standard intervals throughout the year.

### Participants and procedures

We invited third-grade pupils (aged 8–9) from two private elementary schools in a metropolitan area in the Philippines to participate in our study. To have an equal participant distribution, we only recruited students from three sections in third-grade classes per school. The eligibility criteria of our study included students who voluntarily accepted to participate, have parental permission (informed consent was required), and with access to an Internet-connected device at home. The average class size of both schools was 30 pupils per section. All participants (*n* = 180) were eligible and participated. Before commencing the study, we oriented all teachers and parents on their specific roles. We performed the intervention assignment by school level, with all participating students engaging in either the 360V or REGV intervention.

Before joining a VFT, we asked students to complete a ten-item content recall test (pre-test). We administered the same assessment after the VFT (post-test) as well as a VFT experience questionnaire (VFT-XP). In the first round of the experiment (Q1), both groups attended a VFT, with G1 engaging in the 360V (experimental group) and G2 engaging in the REGV (control group). We replicated this experiment in Q2 but the groups switched roles, with G1 serving as a control group (using REGV) and G2 as the experimental group (using 360V). We administered a similar set of content recall tests aligned with the contents of this quarter. The experiments were replicated again for Q3 and Q4, switching the group roles accordingly and adjusting the assessments to align with the VFT content (see Table 1).

### Interventions and videos

**Fig. 1:**
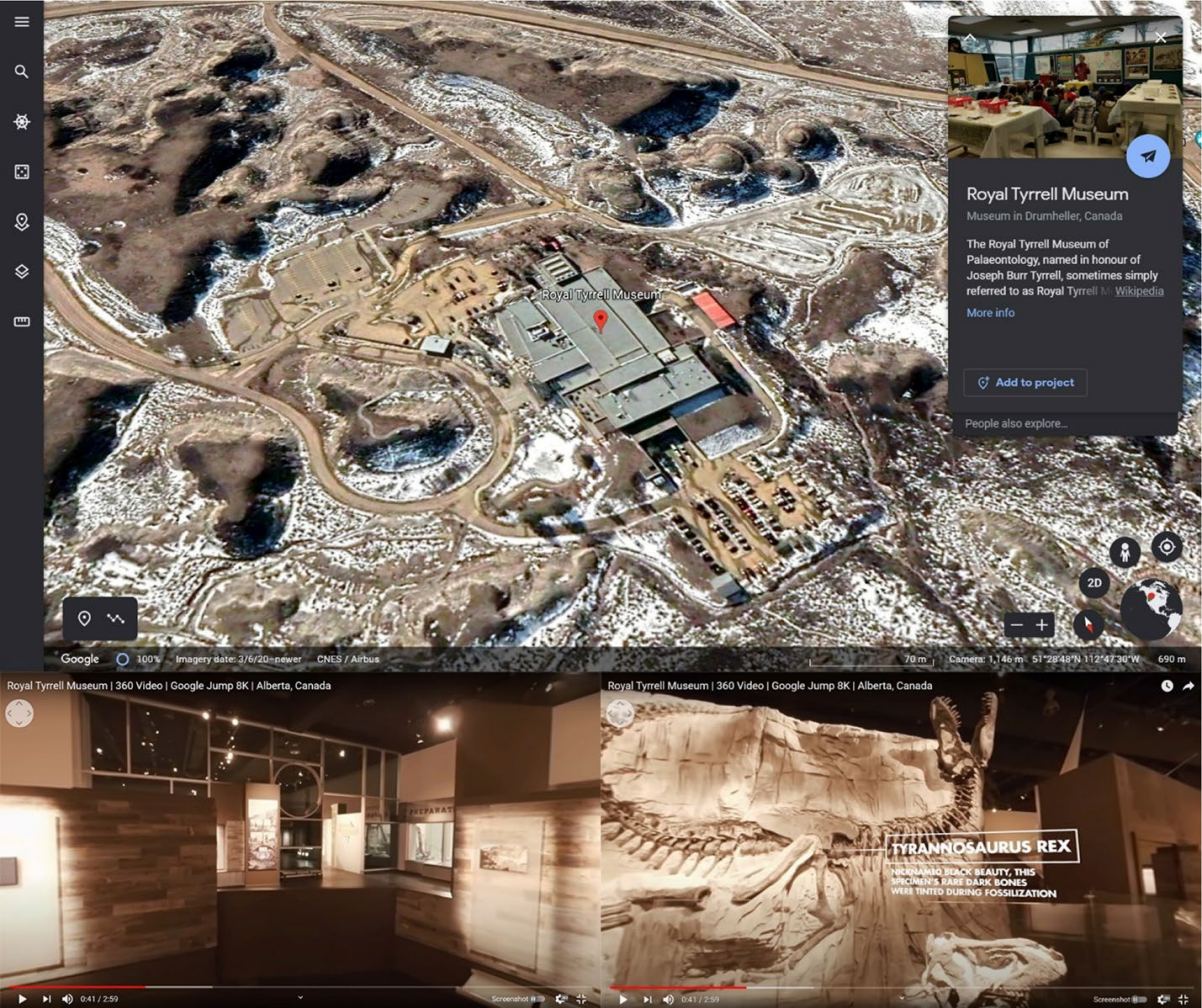
Tour recording of a virtual field trip to the royal Tyrell Museum, Alberta, Canada

The foci of the VFTs in our study are common locations of PFTs, including farms and factories (Q1), museums and galleries (Q2), zoos and wildlife parks (Q3), and historical sites and landmarks (Q4). Since it was impossible to shoot videos during the study, we relied on a combination of 360° videos from various online video sharing platforms for indoor shots and custom tour recordings using Google Earth for outdoor shots (see Fig. 1). We edited and assembled all video materials in post-production using *Adobe Premiere Pro*. Similar to the discussions in PFTs, we added narration to share meaningful stories, interesting facts, and the VFT lessons. Although it is possible to transform the 360° videos and create VR videos, we purposely chose not to because the converted videos require a wearable VR headset to view. The mean runtime of video clips was one hour and two minutes. We uploaded all videos to Google Drive and shared the links with the cooperating teachers and parents during the orientation. We encouraged downloading the videos ahead of time, especially for those with slow internet connection.

### Instrument development

We developed and used two instruments to evaluate the interventions: multiple choice tests (with four possible answers) and the VFT-XP questionnaire. The purpose of the assessment tests was to evaluate the content recall of pupils before and after each VFT. As a team of two academic heads and six primary teachers, we engaged in a Modified Delphi method to develop eight content recall tests with ten items each (one per quarter with two variations each for pre- and post-test). The team determined the salient content, verified it was aligned with the videos, and ensured the language used in the tests was age appropriate for third-grade level students. On the other hand, the VFT-XP was intended to measure the virtual experience of pupils in a VFT. Since VFT is still in its infancy stage and there is no thorough investigation yet on factors that influence the success of a VFT, we created a custom instrument based on a variety of related studies. The final VFT-XP questionnaire (see “Appendix”) has six subscales: attitude (Orion & Hofstein, 1994), perceived usefulness (Arents et al., 2021), inquiry (Behrendt & Franklin, 2014), involvement (Han, 2020), video engagement (Dobrian et al., 2011), and virtual guide (Lavie Alon & Tal, 2015). The Cronbach’s alpha values for the internal consistency of all constructs (attitude = 0.73, perceived usefulness = 0.71, inquiry = 0.81, involvement = 0.87, video engagement = 0.85, and virtual guide = 0.83) were greater than 0.70, indicating acceptable reliability (Taber, 2018).

### Statistical analyses

We analyzed the collected data using IBM SPSS Statistics 26.0. Customarily, a two-way mixed analysis of variance (ANOVA) should be used to determine if there are interactions between the groups over time, e.g., whether pupils’ content recall scores changed over time depending on their intervention (REGV or 360V). However, since the participants switched roles per quarter, it violates one of the basic assumptions of this statistical test: there should be no relationship between the observations in each category of the between-subjects factor (Seltman, 2018). Consequently, we employed a combination of parametric and non-parametric tests (for data that violates the normality assumption) for our analysis. We used paired-sample t-tests for our within-group analysis per quarter to determine whether there was a significant difference in content recall before and after VFTs (pre- vs. post-test). In addition, we used independent-samples t-tests and Mann–Whitney *U* tests for between-group analyses (G1 vs. G2) of both content recall and VFT-XP per each quarter and group. Lastly, we used one-way repeated measures ANOVA and Friedman Tests to determine whether there was a significant difference in content recall and VFT-XP per group across four-time points (Q1 vs. Q2 vs. Q3 vs. Q4).

## Results

### Content recall

We conducted a series of paired-sample *t*-tests to determine whether there was a statistically significant mean difference in content recall scores before and after VFTs per each quarter. No outliers were detected through a visual inspection of boxplots, and the assumption of normality was not violated as assessed by Shapiro–Wilk’s test (*p* > 0.05). As shown in Table 2, the results of our paired-samples t-tests revealed only one significant increase in content recall (Q3: Zoos and Wildlife Parks; *p* = 0.043). However, there was an interesting pattern in the results: REGV groups had higher mean content recall scores during pre-tests but were surpassed by 360V groups during post-tests for all quarters. This finding indicates that the increase in content recall scores of 360V groups after VFTs were consistently higher compared to REGV groups. On the first VFT (Q1), the mean content recall scores of G1 had increased by 1.20 points (95% CI 0.910 to 1.490; *t* = 8.227) while G2 had increased only by 0.82 points (95% CI 0.485 to 1.137; *t* = 4.942). After switching their roles (Q2), G2 had increased by 1.12 points (95% CI 0.530 to 1.114; *t* = 5.592) while G1 had only increased by 0.82 points (95% CI 0.821 to 1.423; *t* = 7.411). We observed similar findings on the succeeding quarters where G1 (2.20 points; 95% CI 1.822 to 2.578; *t* = 11.554) had a higher increase on mean content recall score compared to G2 (0.97 points; 95% CI 0.705 to 1.228; *t* = 7.341) during Q3, and G2 (1.76 points; 95% CI 0.466 to 1.023; *t* = 7.815) had a higher increase on mean content recall score compared to G1 (0.93 points; 95% CI 0.671 to 1.196; *t* = 7.070) during Q4. The group with a 360V intervention had always a higher increase in mean content recall score after each VFT.

**Table 2:** Paired t-test results of before and after VFTs

Quarter	Group	Treatment	Pre-test	Post-test	Difference	*t*	*p*
Q1	G1	360V	6.84 ± 0.90	8.04 ± 1.02	1.20 ± 1.38	8.227	0.985
	G2	REGV	7.04 ± 1.04	7.86 ± 1.06	0.82 ± 1.44	4.492	0.152
Q2	G1	REGV	6.06 ± 1.01	6.88 ± 0.99	0.82 ± 1.81	5.592	0.924
	G2	360V	5.89 ± 0.95	7.01 ± 1.03	1.12 ± 1.25	7.411	0.715
Q3	G1	360V	6.03 ± 0.92	8.23 ± 1.43	2.20 ± 1.56	11.554	0.043*
	G2	REGV	6.62 ± 0.98	7.99 ± 0.91	0.97 ± 1.39	7.341	0.692
Q4	G1	REGV	5.91 ± 7.84	6.84 ± 1.04	0.93 ± 1.25	7.070	0.799
	G2	360V	5.86 ± 1.01	7.62 ± 0.91	1.76 ± 1.33	7.815	0.618

Data are mean ± standard deviation. *Significant

**Fig. 2:**
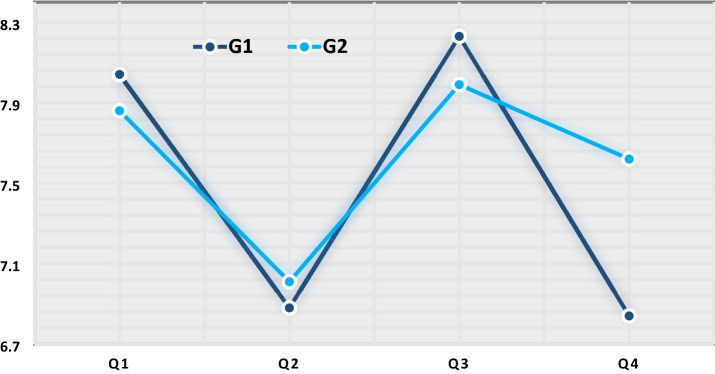
Between-group comparison of post-test content recall scores

We also conducted a series of independent-sample *t*-tests to determine whether there was a statistically significant mean difference in post-test scores between the groups (see Fig. 2). There were no outliers in the data as assessed by visual inspection, the assumption of normality was not violated as assessed by Shapiro–Wilk’s test (*p* > 0.05), and variances were homogeneous as assessed by Levene’s test (*p* values: Q1 = 0.405, Q2 = 0.885, Q3 = 0.309, Q4 = 0.097). The difference in post-test scores between the groups was only significant during Q4 (Historical Sites and Landmarks, *p* = 0.039). We continued our analysis by examining the results of a one-way repeated measure ANOVA to determine whether there was a statistically significant difference in post-test scores of each group over the course of four VFTs. While there were no outliers in the data and the assumption of normality was not violated, the assumption of sphericity was only met by G1 (*χ*^2^[5] = 8.58, *p* = 0.127) but not met in G2: (*χ*^2^[5] = 21.02, *p* = 0.000), as assessed by Mauchly’s test of sphericity. Therefore, we applied a Greenhouse–Geisser correction for G2 (*ε* = 0.699). We found that VFTs elicited statistically significant changes in content recall over time for G1 (*F*[3, 267] = 50.84, *p* < 0.05, partial *η*^2^ = 0.36) and G2 (*F*[3, 267] = 135.70, *p* < 0.05, partial *η*^2^ = 0.60). This finding indicates that switching the intervention affects the assessment scores and this outcome is likely caused by the specific intervention assigned to the group.

### VFT experience

**Fig. 3:**
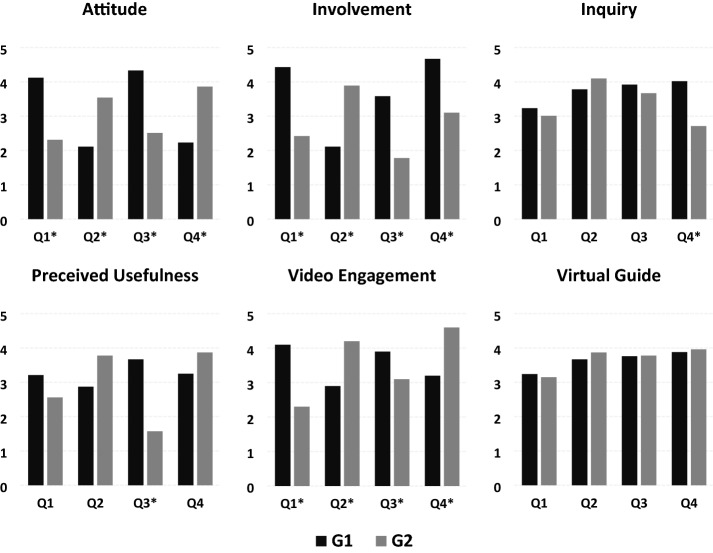
Between-group comparison of VFT-XP mean scores. *Any quarter where mean scores are statistically significant

For the VFT-XP questionnaire, we conducted a series of Mann–Whitney *U* tests to determine if there were differences between the two groups in terms of attitude, perceived usefulness, inquiry, involvement, video engagement, and virtual guide per quarter. During Q1, median scores were only statistically significantly different in terms of attitude (*U* = 2,917, *z* = −3.365, *p* = 0.001), video engagement (*U* = 5,613, *z* = 4.638, *p* = 0.000), and involvement (*U* = 2,123, *z* = −2.292, *p* = 0.001). Similar findings were found on Q2 where median scores were statistically significantly different in terms of attitude (*U* = 3,423, *z* = −1.955, *p* = 0.024), video engagement (*U* = 6,199, *z* = 5.215, *p* = 0.000), and involvement (*U* = 3,339, *z* = 2.118, *p* = 0.019). Meanwhile, median scores during Q3 were statistically significantly different in terms of attitude (*U* = 3,432, *z* = −4.174, *p* = 0.000), perceived usefulness (*U* = 4,784, *z* = 3.967, *p* = 0.004), video engagement (*U* = 4,348, *z* = −3.929, *p* = 0.000), and involvement (*U* = 4,189, *z* = 3.378, *p* = 0.000). Lastly, median scores during Q4 were statistically significantly different in terms of attitude (*U* = 3,243, *z* = −4.238, *p* = 0.000), inquiry (*U* = 2,547, *z* = −3.216, *p* = 0.001), video engagement (*U* = 2,567, *z* = 2.583, *p* = 0.000), and involvement (*U* = 3,624, *z* = −2.368, *p* = 0.028). In Fig. 3, we used the mean scores of VFT-XP across four quarters to show the between-group comparison.

To summarize, only three constructs (attitude, video engagement, and involvement) from the VFT-XP were consistently rated significantly differently by pupils in all quarters. Conversely, the virtual guide was the only factor that was consistently rated not significantly different in all quarters. We also found that pupils’ perceptions of perceived usefulness and inquiry were mixed and rated significantly different during Q3 and Q4, respectively. We continued our analysis by conducting a Friedman test to determine if there were significant differences in VFT-XP constructs over the course of four VFTs. Our results show that engaging in VFTs elicited statistically significant changes over time for G1 in terms of attitude (*χ*^2^(2) = 17.684, *p* = 0.001), involvement (*χ*^2^(2) = 24.149, *p* = 0.000), and video engagement (*χ*^2^(2) = 29.110, *p* = 0.000), and for G2 in terms of attitude (*χ*^2^(2) = 16.256, *p* = 0.000), involvement (*χ*^2^(2) = 18.299, *p* = 0.002), and video engagement (*χ*^2^(2) = 21.926, *p* = 0.042). The remaining constructs did not have significant changes.

## Discussion

### Cognitive effects of going into a VFT

The result of within-group analyses indicated that the increase in content recall scores of 360V groups after VFTs was consistently higher compared to REGV groups at all time points. Although, pupils from REGV learned as much except during a VFT on Zoos and Wildlife Parks (Q3), which echoes existing studies where no significant differences were found in the learning outcomes (Araiza-Alba et al., 2021; Arents et al., 2021). We partially attribute the consistent increase in content recall scores to 360° videos because of its capability to create authentic learning opportunities and engage pupils more realistically. The inclusion of such realism requires less active imagination of the world during the knowledge acquisition process, thereby reducing cognitive demands. Further, the engagement provided by a VFT allows learners to take more active self-responsibility for their learning process. Although not to the full extent, 360° videos provided an opportunity for pupils to make their own discoveries. Unlike existing studies (e.g., the VFT to Corinthian Gulf; Evelpidou et al., 2021) that only used Google Earth (outdoor scenes), one advantage of our study is that it incorporated 360° videos to show indoor shots of museums, farms, zoos, and others. We speculate that by allowing pupils to virtually travel from any starting point (e.g., school) to the actual field trip locations (e.g., zoo) including its indoor views, the experience becomes almost similar to traditional field trips. Despite 360° videos being less immersive than 360° videos in a VR platform, our experiment implies that 360° videos can still elevate the engagement and sense of immersion and authenticity while pupils are exploring in the virtual realm, which reiterates the findings of Argyriou et al. (2020).

### The quality of experience in a VFT

Our results also show that VFTs elicited statistically significant changes in VFT experience for both groups over time. Meaning, that at all VFT sessions, the experience of each group varies depending on the allocated treatment (REGV or 360V). Nevertheless, only attitude, video engagement, and involvement from the VFT-XP were consistently rated significantly different by pupils in all quarters, while the virtual guide was the only factor that was consistently rated not significantly different. To elaborate, pupils have a more positive attitude towards VFT and are more likely to attend another session willingly when 360V is the mode of virtual excursion. As demonstrated by Orion and Hofstein (1994), the fun factor is a determinant of a positive attitude towards field trips, and the immersive view functionality offered by 360V may have contributed to the amusement of pupils. This affective state could also explain why they were more engaged and involved since they can navigate the real world even in the comfort of their homes as compared to simply watching static videos. Although pupils may have been overwhelmed by the novelty effect of a VFT as mentioned in other studies (e.g., Zhao et al., 2021), the longitudinal nature of our experiment settles this uncertainty. Upon examination, these positive affective outcomes seem to be in a tug of war with the cognitive outcomes. That is, although not significant, the virtual guide factor was rated higher when REGV is the VFT mode. This finding suggests that pupils cannot focus on teachers’ narration on 360° videos because they were too engaged with navigating the videos. Since they were not attentive to the information being narrated throughout the videos, it may have affected their content recall scores where the assessments were based on. This deviation is further supported by our mixed findings on the inquiry construct, which measure the effect of convincing pupils to want to study and learn more.

### Implications, limitations, and future research

From a theoretical standpoint, our study contributes to the literature on exploiting 360° videos for educational purposes and implementing VFTs as a viable alternative to PFTs. Previous studies have emphasized that VFTs are not a replacement but only a supplement to PFTs that provide necessary pre-information before students visit an actual location (Seifan et al., 2019) or after an in-person field trip for better recall of experience (Harron et al., 2019). Although we share the same beliefs, the restrictions posed by the COVID-19 pandemic provided us with an opportunity to be the first study to completely replace PFTs with VFTs. Thus, our findings offer new insights and evidence on VFTs that may be valuable in educational policy formation. For instance, schools may finally address potential equity issues by offering a VFT option to students who could not afford to go on field trips. Rather than a once-a-year-only event, teachers could maximize VFTs as an instructional strategy to support teaching and learning both inside and outside the classroom. Parents would not have to worry about their children getting separated, loss, or hurt, especially in field trips in which they cannot chaperone. Finally, students could benefit by having the opportunity to go in any VFT from anywhere at any time.

Despite the positive results of our study, we do not claim that VFTs could be used as a substitute for in-person field trips. A critical factor that is missing from this field trip version is the opportunity for social interaction. According to Behrendt and Franklin (2014), field trips promote social growth by encouraging positive interactions between students, parents, and teachers. While we assumed pupils may have been very interested in the VFT experience, we did not anticipate the potential for internet fatigue due to school shuttering and being overwhelmed with online learning experiences (Garcia, 2022). In addition, one of our concerns was how pupils interacted with the VFT experience. Although we recognized their engagement was independent of the teacher, we were not able to determine how they interacted with the 360° videos, the duration of time on specific content, or the return to a particular timestamp for further exploration. Gathering these information requires the integration of an appropriate framework for video viewing behaviors (Kleftodimos & Evangelidis, 2014), educational data mining (El Aouifi et al., 2021) and video analytics (Zhou et al., 2021). Another concern was pupils’ actual perceptions of the experience. While we assume our instrument was aligned with their VFT journey, their experience may have varied based on internet access, technology capacity, and parental guidance. Prior research suggests that although 360° videos are more immersive than traditional 2D videos, they are still less immersive than 360° videos in a VR platform (Rupp et al., 2019). Future researchers should compare standard 360° videos and 360° videos in VR environments to determine whether the level of immersiveness is translatable to the quality of a VFT. We also encourage researchers to replicate our experiment at other levels of education. Prior works mainly used VR for conducting a VFT (e.g., Harron et al., 2019).

Although our study addressed the potential obstacle of needing to acquire high-fidelity VR headsets for a wide-scale VFT implementation, it also introduces potential constraints for future adopters. For instance, we relied on available 360° videos since it was impossible to shoot our own because of the COVID-19 pandemic. We found that the dependency on the existing 360° videos is not ideal for a personalized VFT (e.g., selecting the field trip locations and how they are presented in the videos). More importantly, these videos were not deliberately captured for VFT purposes and may need to be reshot to meet the curriculum needs. Therefore, special camera equipment capable of capturing 360° videos is required. The expertise of instructional designers is also recommended to ensure that the content area is covered and the instruction is developmentally appropriate for the intended audience. VFT also demands ample time to design the experience, create video materials, and ensure that everything is compliant with specific educational objectives. As echoed by existing studies (Hudak, 2003; Kisiel, 2006; Knapp & Barrie, 2001; Nadelson & Jordan, 2012), a well-organized field trip yields successful cognitive, affective, and psychomotor outcomes. On the positive side, the design and development of VFT using 360° videos can become a professional development experience (e.g., learning content design, videography, and video editing) for all school administrators and teachers involved.

## Conclusion

Our research addressed the gap in the literature related to the use of 360° videos for VFTs as an alternative to PFTs in primary education. Through four rounds of experimentation, we found that students assigned to the 360V group consistently scored higher than the REGV group, although not significantly. Further, the VFT experience of pupils varies depending on the allocated treatment (i.e., REGV or 360V). By exploring the possibility of substituting PFTs with 360° videos, our study has provided insights for schools, policymakers, administrators, and teachers to design, develop, and deploy VFTs that allow students to gain experience that cannot be duplicated in traditional school experiences. These results call attention to a possible implementation of VFT and continue the long-standing tradition that has been acknowledged as an integral student-centered, interactive educational method.

## Data Availability

The datasets used and/or analyzed during the current study are available from the corresponding author at a reasonable request.

## Appendix: VFT-XP Questionnaire Items

### Attitude

1. I like going on a virtual field trip.
2. I think a virtual field trip is valuable in class.
3. I believe it is good for me to go in a virtual field trip.
4. I have a positive attitude towards virtual field trips.

### Perceived usefulness

1. The use of videos makes it easy to have a virtual field trip.
2. A virtual field trip is as informative as a physical field trip.
3. Going in a virtual field trip is a useful learning experience.

### Inquiry

1. After a virtual field trip, it makes me want to learn more.
2. After a virtual field trip, it makes me wonder about the world around me.
3. After a virtual field trip, it makes me curious about a number of things.

### Involvement

1. I feel like I am physically present at the location.
2. I can immerse in the field trip location virtually.
3. I feel like I am really part of the virtual field trip.

### Video engagement

1. While watching a video, I can completely focus on the content.
2. While watching a video, I am carried away by the experience.
3. While watching a video, I do not notice the time passing by.
4. While watching a video, I pay a lot of attention to the environment.

### Virtual guide

1. I enjoy listening to the stories during a virtual field trip.
2. I can understand the explanation of the virtual guide.
3. The virtual guide makes me more excited and enthusiastic.

## References

[CR1] Alferes, V. R. (2012). *Methods of randomization in experimental design*. Sage Publications. 10.4135/9781452270012

[CR2] Alsiri NF, Alhadhoud MA, Palmer S (2021). The impact of the COVID-19 on research. Journal of Clinical Epidemiology.

[CR3] Araiza-Alba P, Keane T, Matthews B, Simpson K, Strugnell G, Chen WS, Kaufman J (2021). The potential of 360-degree virtual reality videos to teach water-safety skills to children. Computers and Education.

[CR4] Ardisara A, Fung FM (2018). Integrating 360° videos in an undergraduate chemistry laboratory course. Journal of Chemical Education.

[CR5] Arents V, de Groot PCM, Struben VMD, van Stralen KJ (2021). Use of 360° virtual reality video in medical obstetrical education: a quasi-experimental Design. BMC Medical Education.

[CR6] Argyriou L, Economou D, Bouki V (2020). Design methodology for 360° immersive video applications: the case study of a cultural heritage virtual tour. Personal and Ubiquitous Computing.

[CR7] Behrendt, M., & Franklin, T. (2014). A review of research on school field trips and their value in education. *International Journal of Environmental and Science Education*,* 9*, 235–245. 10.12973/ijese.2014.213a

[CR8] Bowker R, Tearle P (2007). Gardening as a learning environment: a study of children’s perceptions and understanding of school gardens as part of an international project. Learning Environments Research.

[CR9] Çaliskan O (2011). Virtual field trips in education of earth and environmental sciences. Procedia Social and Behavioral Sciences.

[CR10] Davies RS, Williams DD, Yanchar S (2008). The use of randomisation in educational research and evaluation: a critical analysis of underlying assumptions. Evaluation and Research in Education.

[CR11] DeWitt J, Storksdieck M (2008). A short review of school field trips: key findings from the past and implications for the future. Visitor Studies.

[CR12] Dobrian, F., Sekar, V., Awan, A., Stoica, I., Joseph, D., Ganjam, A., Zhan, J., & Zhang, H. (2011). Understanding the impact of video quality on user engagement. *ACM SIGCOMM 2011 Conference* (pp. 362–373). 10.1145/2018436.2018478

[CR13] Edmonds, W. A., & Kennedy, T. D. (2017). Within-subjects approach. In *An applied guide to research designs: Quantitative, qualitative, and mixed methods* (2nd ed.). Sage Publications. 10.4135/9781071802779

[CR14] El Aouifi H, El Hajji M, Es-Saady Y, Douzi H (2021). Predicting learner’s performance through video sequences viewing behavior analysis using educational data-mining. Education and Information Technologies.

[CR15] Evelpidou N, Karkani A, Saitis G, Spyrou E (2021). Virtual field trips as a tool for indirect geomorphological experience: a case study from the southeastern part of the Gulf of Corinth, Greece. Geoscience Communication.

[CR16] Garcia, M. B. (Ed.). (2022). *Socioeconomic inclusion during an era of online education*. IGI Global. 10.4018/978-1-6684-4364-4.

[CR17] Han I (2020). Immersive virtual field trips in education: a mixed-methods study on elementary students' presence and perceived learning. British Journal of Educational Technology.

[CR18] Harron, J. R., Petrosino, A. J., & Jenevein, S. (2019). Using virtual reality to augment museum-based field trips in a preservice elementary science methods course. *Contemporary Issues in Technology and Teacher Education*,* 9*(4), 687–707. https://citejournal.org/wp-content/uploads/2019/11/v19i4Science1.pdf

[CR19] Higginsa N, Dewhursta E, Watkinsb L (2012). Field trips as short-term experiential learning activities in legal education. The Law Teacher.

[CR20] Hoisington, C., Sableski, N., & DeCosta, I. (2010). A walk in the woods. *Science and Children*, *48*(2), 27–31. https://foundationsofscienceliteracy.edc.org/wp-content/uploads/2019/07/SC-Vol48-2.pdf

[CR21] Hosticka, A., Schriver, M., Bedell, J., & Clark, K. (2002). Computer based virtual field trips. *EdMedia + Innovate Learning 2002*, 312–316. https://www.learntechlib.org/p/9289

[CR22] Huang, X.-q., & Hong, W. (2017). Live streaming teaching applied in real english classroom. *DEStech Transactions on Social Science Education and Human Science*. 10.12783/dtssehs/aems2017/8278

[CR23] Hudak PE (2003). Campus field exercises for introductory geoscience courses. Journal of Geography.

[CR24] Jitmahantakul, S., & Chenrai, P. (2019). Applying virtual reality technology to geoscience classrooms. *Review of International Geographical Education Online*,* 9*(3), 577–590. 10.33403/rigeo.592771

[CR25] Kisiel J (2006). More than lions and tigers and bears: creating meaningful field trip lessons. Science Activities.

[CR26] Kleftodimos, A., & Evangelidis, G. (2014). Exploring student viewing behaviors in online educational videos. In *2014 IEEE 14th international conference on advanced learning technologies* (pp. 367–369). 10.1109/ICALT.2014.109

[CR27] Knapp D, Barrie E (2001). Content evaluation of an environmental science field trip. Journal of Science Education and Technology.

[CR28] Kola-Olusanya A (2005). Free-choice environmental education: understanding where children learn outside of school. Environmental Education Research.

[CR29] Kosterelioglu, I. (2016). Student views on learning environments enriched by video clips. *Universal Journal of Educational Research*,* 4*(2), 359–369. 10.13189/ujer.2016.040207

[CR30] Lavie Alon N, Tal T (2015). Student self-reported learning outcomes of field trips: the pedagogical impact. International Journal of Science Education.

[CR31] Lebak K (2007). Mediating cultural borders during science field trips. Cultural Studies of Science Education.

[CR32] Nadelson LS, Jordan JR (2012). Student attitudes toward and recall of outside day: an environmental science field trip. The Journal of Educational Research.

[CR33] Noetel M, Griffith S, Delaney O, Sanders T, Parker P, del Pozo Cruz B, Lonsdale C (2021). Video improves learning in higher education: a systematic review. Review of Educational Research.

[CR34] Orion N, Hofstein A (1994). Factors that influence learning during a scientific field trip in a natural environment. Journal of Research in Science Teaching.

[CR35] Pokhrel S, Chhetri R (2021). A literature review on impact of covid-19 pandemic on teaching and learning. Higher Education for the Future.

[CR36] Ranieri, M., Bruni, I., & Luzzi, D. (2020). Introducing 360-degree video in higher education: an overview of the literature. European Distance and E-Learning Network, Timisoara, Romania.

[CR37] Rupp MA, Odette KL, Kozachuk J, Michaelis JR, Smither JA, McConnell DS (2019). Investigating learning outcomes and subjective experiences in 360-degree videos. Computers and Education.

[CR38] Seifan M, Dada D, Berenjian A (2019). The effect of virtual field trip as an introductory tool for an engineering real field trip. Education for Chemical Engineers.

[CR39] Seifan, M., Dada, O. D., & Berenjian, A. (2020). The effect of real and virtual construction field trips on students’ perception and career aspiration. *Sustainability*, *12*(3), 1200. https://www.mdpi.com/2071-1050/12/3/1200

[CR40] Seltman HJ (2018). Two-way ANOVA.

[CR41] Shadish WR, Ragsdale K (1996). Random versus nonrandom assignment in controlled experiments: do you get the same answer?. Journal of Consulting and Clinical Psychology.

[CR42] Skop E (2009). Creating field trip-based learning communities. Journal of Geography.

[CR43] Spicer JI, Stratford J (2001). Student perceptions of a virtual field trip to replace a real field trip. Journal of Computer Assisted Learning.

[CR44] Springer, C., Wehking, F., Wolf, M., & Söbke, H. (2020). *Virtualization of virtual field trips: A case study from higher education in environmental engineering*. Fifteenth European Conference on Technology Enhanced Learning, Heidelberg, Germany.

[CR45] Stainfield J, Fisher P, Ford B, Solem M (2000). International virtual field trips: A new direction?. Journal of Geography in Higher Education.

[CR46] Taber KS (2018). The use of Cronbach’s alpha when developing and reporting research instruments in science education. Research in Science Education.

[CR47] Thönnessen, N., & Budke, A. (2021). The use of digital field trip guides for 'learning on-site' and 'virtual excursions' in a covid-19 world. In R. E. Ferdig & K. E. Pytash (Eds.), *What teacher educators should have learned from 2020*. Association for the Advancement of Computing in Education. https://www.researchgate.net/publication/350153629

[CR48] Trochim, W. M., & Donnelly, J. P. (2008). *The research methods knowledge base*. Atomic Dog Publishing/Cengage Learning.

[CR49] Zhao J, Wallgrün JO, Sajjadi P, LaFemina P, Lim KYT, Springer JP, Klippel A (2021). Longitudinal effects in the effectiveness of educational virtual field trips. Journal of Educational Computing Research.

[CR50] Zhou M, Chen GH, Ferreira P, Smith MD (2021). Consumer behavior in the online classroom: Using video analytics and machine learning to understand the consumption of video courseware. Journal of Marketing Research.

